# Jumping and Landing Kinematics in Spanish Female Soccer Players: A Comparison Between Elite and Non-Elite Athletes

**DOI:** 10.3390/s25041109

**Published:** 2025-02-12

**Authors:** Francisco Javier Robles-Palazón, Alba Aparicio-Sarmiento, María Teresa Martínez-Romero, Mark De Ste Croix, Francisco Ayala

**Affiliations:** 1 Department of Physical Activity and Sport, Faculty of Sport Sciences, Campus of Excellence Mare Nostrum, University of Murcia, 30720 Murcia, Spain; franciscojavier.robles1@um.es (F.J.R.-P.); alba.aparicio.sarmiento@gmail.com (A.A.-S.); francisco.ayala@um.es (F.A.); 2 Sports and Musculoskeletal System Research Group (RAQUIS), University of Murcia, 30100 Murcia, Spain; 3 Centre for Human Movement and Rehabilitation, School of Health and Society, University of Salford, Salford M6 6PU, UK; 4 School of Education, Sport and Applied Sciences, University of Gloucestershire, Gloucester GL2 9HW, UK; mdestecroix@glos.ac.uk

**Keywords:** associated football, women, knee injury, valgus, tuck jump, injury risk

## Abstract

Landing from a jump has been identified as a common situation of increased risk in sport and the tuck jump assessment (TJA) has been proposed for a comprehensive examination of landing mechanics. However, group-specific data on female athletes are limited. The purpose of this study was to examine the movement mechanics during a TJA in Spanish female soccer players and to explore potential differences between players of different performance levels. A total of 96 (elite and non-elite) female soccer players performed a TJA, and a rater visually graded the technique using the modified 10-item scoring system (0, 1, or 2 for “none”, “small”, or “large” flaws). Descriptive statistics were calculated. The association between the flaws and performance groups was assessed using the chi-square test. Almost 90% of all players involved were categorized with small and large flaws for the item “Lower extremity valgus at landing”. The proportion of players categorized with technical flaws was also high for “Foot contact timing not equal” (85%) and “Does not land in same footprint” (82%). Differences between elite and non-elite players were only found for “Foot placement not parallel” and “Excessive landing contact noise” (*p* < 0.008). These results reveal the importance of implementing training programs to reduce jumping and landing deficits in female soccer players, independently of the players’ level of performance.

## 1. Introduction

The participation of women in soccer has experienced a remarkable increase on a global scale, with significant growth observed at both amateur and professional levels. A report published by the Union of European Football Associations (UEFA) in 2017/2018 documented that the number of registered professional and semi-professional female players in UEFA’s member associations more than doubled in 4 years from 1680 in 2013 to 3572 in 2017 [1]. In a further report compiled by the Fédération Internationale de Football Association (FIFA) in 2018, it was estimated that around 30 million participants globally were women and showed an ambition to increase the participation of girls and women in the sport to 60 million by 2026 [2]. Spain mirrors this global trend, marked by the establishment of professional women’s soccer leagues, the competitive success of Spanish teams in international arenas, and heightened media visibility. In fact, the participation of women in Spain has increased by 25% in adult soccer categories in the last five seasons (it has almost doubled in the case of professional players, from 670 to 1233 participants) [3,4]. Consequently, Spanish women’s soccer has solidified its position as a significant force on the international stage, with more and more female players taking advantage of the many benefits of playing this sport every year [5,6].

However, the rise in participation has also led to an increase in the number of soccer-related injuries in women. Recent meta-analyses have reported significant injury rates in female soccer players, with incidences of around 6 injuries per 1000 h of exposure and that can reach up to 19–20 injuries per 1000 h in matches [7,8,9]. Knee injuries represent a substantial proportion of total injuries, accounting for more than 20% of all injuries sustained during training and matches [10]. Furthermore, Materne et al. [11] reported that knee injuries in general, and anterior cruciate ligament (ACL) tears in particular, contribute the highest injury burden in terms of time lost from soccer participation and the need for medical intervention. The main mechanisms underlying knee injuries include non-contact or indirect contact actions, with landing after a jump identified as one of the high-risk situations for these injuries in soccer [12]. To mitigate the risk of knee injuries, it is then essential to identify at-risk athletes through screening protocols for altered mechanics during jumping and landing actions.

One prominent and practical method for a comprehensive examination of landing mechanics is the Tuck Jump Assessment (TJA) [13]. The TJA is widely regarded as a clinician-friendly screening tool, capable of assessing movement patterns linked to injury risk during jumping and landing tasks in an accessible, simple, and cost-effective environment [14]. Far from complex and sophisticated motion capture systems, the TJA offers immediate feedback on movement quality, providing coaches and practitioners with valuable practical information for daily use with their athletes. However, and despite its growing application, research using the TJA test to analyze jumping and landing mechanics in female players remains scarce, especially in Spanish women’s soccer. Much of the existing literature is based on mixed-sports [15,16,17] or male-dominant cohorts [18,19,20], limiting the generalizability of findings to female populations. This research gap highlights the need for specific data on Spanish female soccer players. Research focused on elite and non-elite female soccer players could provide information to tailor training, optimize athlete development, and enhance injury prevention measures throughout the different levels of performance.

Therefore, the purposes of this research were (1) to examine the movement mechanics during a TJA test in Spanish female soccer players and (2) to explore potential differences between players at different performance levels (elite vs. non-elite).

## 2. Materials and Methods

### 2.1. Design

A cross-sectional observational design was employed to describe the jumping and landing kinematics during a TJA protocol among female soccer players. All the assessments were carried out during the preseason periods (September) of the years 2021 and 2022.

The testing session was divided into two different parts: the first part was designed to evaluate the players’ demographic and anthropometric measures; the second part was used to collect data for the TJA test. A 20-min standardized dynamic warm-up was performed before the TJA data collection, which included whole body exercises emphasizing dynamic mobilization and gradually progressing in intensity [21]. Participants were asked to wear shorts that extended to about mid-thigh, allowing for clear visibility of leg movement. The qualitative kinematic analysis was retrospectively conducted through 2-D video recordings.

### 2.2. Participants

A total sample of 96 Spanish female soccer players from seven different soccer clubs completed this study. The players competed in the Spanish women’s professional soccer league (*n* = 18) and in other lower tiers (*n* = 78) and were free of injuries and delayed onset muscle soreness (DOMS) at the time of testing (self-reported). Descriptive statistics for participants’ characteristics are presented in Table 1. All participants were instructed to avoid engaging in vigorous exercise for at least 48 h before the testing session. The experimental procedures applied in this study adhered to the principles outlined in the Declaration of Helsinki and were approved by the Ethics and Scientific Committee of the University of Murcia (Spain) (ID: 2424/2019). Participants provided written informed consent prior to the commencement of the study.

### 2.3. Procedures

#### Tuck Jump Assessment (TJA)

Tuck jumps were performed in place for a period of 10 consecutive seconds, following the protocol described by Myer et al. [13]. Participants were verbally instructed to initiate with a countermovement, followed by consecutive vertical jumps performed as high as possible while simultaneously pulling their knees up towards their chest [13]. Players were asked to land in the same footprint, minimizing ground contact time. Two vertical strips, positioned 35 cm apart and connected by a horizontal line to form an H-shape, were drawn to ensure proper foot positioning [20]. The jumps were conducted on the soccer field, with players wearing their normal footwear used during competition. A demonstration of performance was also given if they requested further assistance or did not perform the test correctly after the verbal instructions. Each player performed three consecutive repetitions to familiarize themselves with the test, followed by a single trial of the TJA.

The TJA was recorded using 3 video cameras on tripods, one aligned in the frontal plane and two aligned on both sides in the sagittal plane, sampling at 120 frames per second and using a high-definition resolution (Lumix DMC-FZ200, Panasonic, Osaka, Japan). All video cameras were positioned 5 m from the landing area for full coverage of the jumps and landing. A sport scientist with previous training in kinematic assessment and more than 3 years of experience in this specific landing analysis independently assessed the videos of the tuck jumps. The videos were analyzed with freely available software (Kinovea 0.8.15, USA) that allowed for slow motion and frame-by-frame advancement. The rater was permitted to replay the videos as many times as necessary to assign a score for the test. Each participant’s TJA was scored using the 10 criteria for flaws. The procedure and scoring system followed the modified version proposed by Fort-Vanmeerhaeghe et al. [22]. That is, if participants demonstrated a technical flaw, then they scored 1 (small flaw) or 2 (large flaw) [22]. Conversely, if participants did not display the flaw, a score of 0 was assigned for the respective category. For a movement to be considered dysfunctional, the specific technique error needed to occur at least twice during the 10-s test [13,22]. The overall TJA score, ranging from 0 to 20, was calculated by summing the flaws identified across the 10 assessment criteria. A higher total score indicated worse performance on the TJA. The rater also registered the total number of jumps performed during the 10-s test duration and the time required for the assessment of each athlete.

### 2.4. Statistical Analysis

Descriptive demographic statistics were calculated as means (*M*) with standard deviations (SDs). Differences in demographic characteristics were assessed with independent *t*-tests. Descriptive statistics, including median, interquartile range, mode, and proportions, were also calculated for all flaws. Differences between competitive groups (elite [RFEF 1st Division] vs. non-elite [other lower tiers] players) for each of the 10 flaws were analyzed by means of the chi-square test. Intra-rater reliability was also evaluated using a sub-sample of 10 participants analyzed at two time points separated by one month. To assess overall agreement between the two evaluations across all items, a global weighted Cohen’s kappa coefficient (*κ*) was calculated. The kappa analysis used quadratic weighting to account for the ordinal nature of the scoring system (0 = no flaw, 1 = small flaw, 2 = large flaw). The scores were analyzed for percentage of exact agreement (PEA) as well.

All statistical analyses were conducted using the JASP computer software (version 0.13.1) and the Statistical Package for Social Science (IBM Corp.; IBM SPSS Statistics for Windows, version 20.0, Armonk, NY, USA). An alpha level of 0.05 was set to determine statistical significance.

## 3. Results

A total of 96 female soccer players participated in this study, with 18 competing in the highest Spanish women’s professional soccer league and 78 playing in other lower tiers. The analysis of the participants’ characteristics revealed that the players in both groups were comparable in height, weight, and BMI, but differed significantly in age (*p* = 0.012), as observed in Table 1.

**Figure 1:**
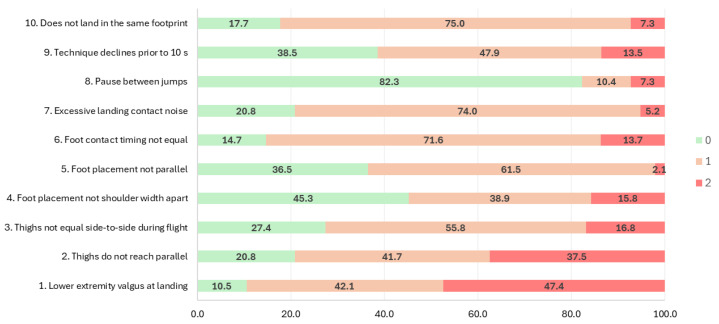
Proportion of players exhibiting each TJA flaw. Note: 0, 1, or 2 for “none”, “small”, or “large” flaws.

Values ranged from 0 to 2 for all technical flaws and for the total sample (including both elite and non-elite athletes), with item 1 “Lower extremity valgus at landing” presenting the worst most repeated value among all items analyzed (47.4% of players received a score of 2). In fact, almost 90% of all players involved in this study were categorized with small and large technical flaws for item 1, “Lower extremity valgus at landing”. The proportion of players categorized with technical flaws was also high for the items “Foot contact timing not equal” (85%) and “Does not land in same footprint” (82%) (Figure 1).

Differences between elite and non-elite players were only found for the items “Foot placement not parallel” and “Excessive landing contact noise” (*χ*^2^ = 10.020–26.303; *p* < 0.008), with non-elite athletes showing a higher prevalence of these technical flaws than their elite counterparts (Table 2). Non-elite players also obtained a greater average number of technical flaws on the TJA total score than elite players (non-elite = 8.8 ± 3.1 vs. elite = 7.1 ± 2.6; *p* = 0.027), while no differences were reported for the total number of jumps between both cohorts of athletes (an average of 15.5 jumps during the 10-s TJA test).

Good agreement was found between the two evaluations for all items and observations (*κ* = 0.728; PEA = 88%, ranged from 80 to 100%).

## 4. Discussion

The present study aimed to examine the movement mechanics during a TJA test in Spanish female soccer players and to explore potential differences between players at different performance levels (elite vs. non-elite). The main findings revealed a different prevalence of technical flaws in the total sample, as well as differences in two landing mechanics between elite and non-elite players. These results have practical implications for injury prevention and performance optimization in female soccer players.

### 4.1. Most Frequent Jumping and Landing Flaws

The analysis of the total sample revealed that the most frequent technical flaws during the TJA were “Lower extremity valgus at landing”, “Foot contact timing not equal”, and “Does not land in the same footprint”. Among these, “Lower extremity valgus at landing” was the most prominent, with nearly 90% of players presenting this flaw, of which 47% obtained a score of “2” (large flaw). This high prevalence is consistent with the results of recent research in female youth handball players, where valgus at landing has also been identified as one of the most common and severe technical flaws [23]. Excessive valgus can increase the strain on the medial collateral ligament (MCL) and anterior cruciate ligament (ACL) due to altered knee joint loading during landing [24,25]. Therefore, the frequency of this undesirable movement pattern highlights the need to integrate neuromuscular training programs that emphasize knee alignment control in Spanish female soccer players. Previous research has shown that improvements in female athletes’ landing mechanics can be observed with as little as 4 to 6 weeks of intervention [26,27]. Training programs aimed at reducing the likelihood of valgus collapse should include targeted exercises to improve hip abductors and external rotators strength, posterior chain activation, and dynamic stability (such as single-leg squats, Nordic hamstring exercise, resisted band exercises, and plyometric drills with feedback on knee position) [28,29,30].

Similar to “Lower extremity valgus at landing”, “Foot contact timing not equal” and “Does not land in the same footprint” were also frequently observed, with 85% and 82% of players showing these flaws, respectively. These findings are noteworthy since asynchronous foot contact and landing instability can increase ground reaction forces, thereby increasing the risk of knee and ankle sprains, which are commonly reported injuries in female soccer players [7,8,9]. Leg dominance and core dysfunction have been proposed as the potential modifiable risk factors for those athletes identified with deficits for the “Foot contact timing not equal” and “Does not land in the same footprint” criteria, respectively [31]. It would, therefore, be advisable to apply lower-extremity plyometric training to improve interlimb symmetry during dynamic tasks, along with trunk and hip training exercises to improve core control in athletes detected with these two TJA flaws [31]. In contrast, the item “Pause between jumps” was the least frequent, with only 18% of players showing this jumping and landing flaw. While this criterion may still contribute to injury risk, it is generally considered less critical than dynamic valgus and foot contact issues. The relationship between this and injury risk may be indirect, affecting fatigue and neuromuscular coordination [15,31,32].

### 4.2. Elite vs. Non-Elite Players

The second objective of this study was to compare the movement mechanics of elite and non-elite Spanish female soccer players. Overall, elite players exhibited better landing patterns for the TJA than non-elite players, with significant differences observed for the mean total score between both competitive levels (7.1 vs. 8.8 for elite and non-elite, respectively). This finding is unsurprising as elite players typically experience higher training intensity and loads and benefit from superior resources compared to their non-elite counterparts [33]. These resources include enhanced access to technical, strength, and stability training programs, as well as support from expert coaches and physical trainers who can implement targeted interventions. Such advantages likely contribute to improved physical conditioning and a greater capacity to control dynamic movements, and thus to a lower frequency and/or magnitude of technical flaws during the TJA task. In any case, despite the statistical significance, this difference in overall TJA scores (1.7 points on a 20-point scale) may not represent a substantial variance from a practical perspective. Given that players in both groups exhibited a high prevalence of technical flaws associated with potential injury risk (such as valgus, asymmetric contact timing, and failure to land in the same footprint), it could be suggested that interventions targeting common risk profiles, rather than focusing on competitive level, may be more effective in mitigating injury risk and enhancing overall movement quality across all players.

When analyzing the prevalence of each individual flaw according to the players’ performance level, our results only revealed significant differences for 2 of the 10 TJA items: “Foot placement not parallel” (*p* < 0.001) and “Excessive landing contact noise” (*p* = 0.007). Regarding “Foot placement not parallel”, 76% of non-elite players demonstrated this flaw, compared to only 11% of elite players. As mentioned above during the discussion on the risk implications of criterion 6 (“Foot contact timing not equal”), this flaw can also negatively affect ground reaction forces and result in asymmetrical loading, which may increase the risk of unilateral lower extremity injuries in the non-elite female cohort [34]. In fact, side-to-side differences in knee load up to six times greater have been observed in ACL-injured females compared to their uninjured peers [35]. Similarly, 85% of non-elite players scored 1 or 2 for the item “Excessive landing contact noise” compared to 55% of elite players. Excessive landing noise could be indicative of stiffer landing mechanics, which are often associated with reduced hip, knee, and ankle flexion angles [36]. These landing mechanics increase the vertical ground reaction forces transmitted to the lower extremities, potentially increasing the risk of injury in lower extremity joints [37]. Myer et al. [29] referred to this risk factor as “quadriceps dominance”, characterized by an imbalance in strength, recruitment, and coordination between the knee extensors and flexors. The observed differences between groups in our study may be indicating a tendency of non-elite players to preferentially activate the quadriceps relative to the hamstrings during the TJA, so athletes identified with this flaw should perform exercises aimed at improving deep knee flexion and posterior chain strength [31].

A noteworthy consideration when interpreting the differences found in movement mechanics between elite and non-elite players is the significant difference in age between both groups. Age-related variations may influence jumping and landing mechanics, potentially contributing to older players demonstrating greater neuromuscular control and strength. However, it should be noted that all participants in this study were older than 14 years and thus likely classified as post-pubertal (peak height velocity estimated to occur around 12 years of age for girls [38]). Consequently, the impact of age differences on movement patterns in this cohort is expected to be less prominent compared to other factors, such as sport experience, training exposure and quality, and physical conditioning. Nonetheless, future studies should consider controlling for age when comparing different performance groups to more precisely isolate the influence of training and competition levels.

Although other flaws, such as “Lower extremity valgus at landing”, “Foot contact timing not equal”, and “Does not land in the same footprint”, were prevalent in both groups, no statistically significant differences were found between elite and non-elite players for them (*p* > 0.05). This similarity could be attributed to the nature of the TJA, which involves a series of repetitive, high-intensity jumping movements performed over a continuous 10-s period [13,22]. Unlike isolated landing tests (e.g., CMJ, DVJ), the TJA demands sustained neuromuscular coordination and control, potentially exposing players of both elite and non-elite levels to comparable technical challenges in movement execution.

### 4.3. Strengths and Limitations

This study highlights the relevance of the TJA as a screening tool to detect potential injury risk factors and identify female athletes in need of specific corrective programs. The simplicity and cost-effectiveness of the TJA (less than 1–2 min to complete the real-time analysis per athlete; less than 5 min to complete the retrospective video analysis) make it a feasible option for coaches and practitioners to assess landing mechanics in daily training environments. By identifying specific technical flaws, practitioners can tailor training interventions that target the underlying movement deficiencies.

However, this study also has some limitations that should be acknowledged. First, the TJA was assessed using 2D video analysis rather than 3D motion capture, which limits the assessment of movement in the transverse plane. However, 2D analysis remains a valid, cost-effective, and practical option for screening in applied sports settings. Second, the rater was not blinded to players’ competitive level, which may have introduced rater bias. Nevertheless, the rater’s extensive experience in assessing landing mechanics and the use of a well-established scoring system are likely to have minimized this bias. Third, the relatively small number of elite players (*n* = 18) included in this study could have limited the statistical power to detect more differences between elite and non-elite groups. Future research should consider larger sample sizes of elite players to enhance the generalizability and robustness of the findings. Finally, another limitation was the lack of longitudinal tracking of injury incidence following the TJA assessment. Future studies should aim to establish a direct link between technical flaws in the TJA and the subsequent risk of lower extremity injuries in Spanish female soccer players. This would help determine the predictive value of TJA flaws in identifying players at risk for injury.

## 5. Conclusions

A high prevalence of key technical flaws during the TJA test is present in Spanish female soccer players, with “Lower extremity valgus at landing” being the most frequent flaw. Differences in movement mechanics between elite and non-elite players were only found for the items “Foot placement not parallel” and “Excessive landing contact noise”. These findings support the integration of neuromuscular and plyometric training in injury prevention programs for female soccer players at all levels of competition. Addressing these technical flaws through specific training interventions may contribute to a reduction in lower extremity injury risk, particularly for knee and ankle ligament injuries.

## Figures and Tables

**Table 1:** Participants’ characteristics (mean ± standard deviation) by performance group.

	Elite (*n* = 18)		Non-Elite (*n* = 78)		*p*-Value
Age (y)	22.6	±5.6	19.5	±4.4	0.012 *
Height (cm)	163	±5.2	161.7	±5.8	0.422
Weight (kg)	57.3	±6.7	58.4	±9.1	0.667
BMI (kg·m^−2^)	21.5	±1.6	22.3	±3.3	0.354

Note: * Significant differences (*p* < 0.05); y = years; cm = centimeters; kg = kilograms; m = meters.

**Table 2:** Comparison of the TJA flaws by performance levels (elite vs. non-elite players).

Tuck Jump Assessment Flaws	Elite (*n* = 18), %	Non-Elite (*n* = 78), %	*x* ^2^	*p*-Value
Score				
1. Lower extremity valgus at landing (F)				
0	17.6	9.0	4.856	0.088
1	58.8	38.5		
2	23.5	52.6		
2. Thighs do not reach parallel (L)				
0	27.8	19.2	3.446	0.179
1	22.2	46.2		
2	50.0	34.6		
3. Thighs not equal side-to-side during flight (F)				
0	23.5	28.2	5.186	0.075
1	76.5	51.3		
2	0.0	20.5		
4. Foot placement not shoulder width apart (F)				
0	52.9	43.6	1.583	0.453
1	41.2	38.5		
2	5.9	17.9		
5. Foot placement not parallel (L)				
0	88.9	24.4	26.303	<0.001 *
1	11.1	73.1		
2	0.0	2.6		
6. Foot contact timing not equal (F)				
0	17.6	14.1	2.056	0.358
1	58.8	74.4		
2	23.5	11.5		
7. Excessive landing contact noise (L)				
0	44.4	15.4	10.02	0.007 *
1	44.4	80.8		
2	11.1	3.8		
8. Pause between jumps (F/L)				
0	88.9	80.8	1.743	0.418
1	11.1	10.3		
2	0.0	9.0		
9. Technique declines prior to 10 s (F/L)				
0	44.4	37.2	0.737	0.692
1	38.9	50.0		
2	16.7	12.8		
10. Does not land in the same footprint (F/L)				
0	5.6	20.5	2.509	0.285
1	88.9	71.8		
2	5.6	7.7		

Note: * Significant differences (*p* < 0.05); F = frontal view; L = lateral view.

## Data Availability

The raw data supporting the conclusions of this article will be made available by the authors upon request.
